# Insular cortex subregions have distinct roles in cued heroin seeking after extinction learning and prolonged withdrawal in rats

**DOI:** 10.1038/s41386-024-01846-x

**Published:** 2024-03-18

**Authors:** Matthew S. McGregor, Caitlin V. Cosme, Ryan T. LaLumiere

**Affiliations:** 1 Interdisciplinary Graduate Program in Neuroscience, University of Iowa, Iowa City, IA 52242 USA; 2 Department of Psychological and Brain Sciences, University of Iowa, Iowa City, IA 52242 USA; 3 Iowa Neuroscience Institute, University of Iowa, Iowa City, IA 52242 USA

**Keywords:** Reward, Operant learning, Extinction, Addiction

## Abstract

Evidence indicates that the anterior (aIC), but not posterior (pIC), insular cortex promotes cued reinstatement of cocaine seeking after extinction in rats. It is unknown whether these subregions also regulate heroin seeking and whether such involvement depends on prior extinction learning. To address these questions, we used baclofen and muscimol (BM) to inactivate the aIC or pIC bilaterally during a seeking test after extinction or prolonged withdrawal from heroin. Male Sprague-Dawley rats in the extinction groups underwent 10+ days of heroin self-administration, followed by 6+ days of extinction sessions, and subsequent cued or heroin-primed reinstatement. Results indicate that aIC inactivation increased cued reinstatement of heroin seeking after extinction, whereas pIC inactivation prevented cued reinstatement. To determine whether these effects were extinction-dependent, we conducted a subsequent study using both sexes with prolonged withdrawal. Male and female rats in the withdrawal groups underwent 10+ days of heroin self-administration, followed by cued seeking tests after 1 and 14 days of homecage withdrawal to measure incubation of heroin craving. In this case, the findings indicate that aIC inactivation had no effect on incubation of heroin craving after withdrawal in either sex, whereas pIC inactivation decreased heroin craving only in males. These findings suggest that the aIC and pIC have opposing roles in suppressing vs promoting cued heroin seeking after extinction and that these roles are distinct from those in cocaine seeking. Moreover, the incubation of craving results suggest that new contingency learning is necessary to recruit the aIC in cued heroin seeking.

## Introduction

Clinical evidence indicates that insular cortex (IC) activity is associated with cue-induced drug craving [1–3] and that IC lesions significantly disrupt nicotine addiction [4]. Consistent with this, work in rodents has found that reversible IC inactivation reduces nicotine self-administration and cued reinstatement, suggesting a role for the IC in promoting nicotine-related behaviors across species [5, 6]. However, there is considerable conflict in the literature as to whether the IC regulates drug seeking across different classes of addictive drugs and whether there is functional heterogeneity within the IC as it relates to these behaviors [7].

Critically, the role of the IC in regulating opioid-related behaviors is understudied. Limited evidence indicates that manipulating either the anterior (aIC) [8] or posterior (pIC) [9, 10] subregion disrupts expression of morphine-induced conditioned place preference (CPP) in rodents. These subregions, which are conserved across species, are thought to be functionally distinct, reflecting differences in the degree of connectivity with corticolimbic structures and thalamic sensory nuclei [11]. Briefly, the pIC is more strongly connected with thalamic sensory nuclei and is considered primary interoceptive cortex, whereas the aIC is more connected with corticolimbic structures and may have higher-order functions related to subjective internal state [12, 13]. Nonetheless, it remains unclear whether the aIC and pIC have distinct roles in drug-related behaviors, largely due to a lack of studies investigating the pIC. Although no prior work has investigated the pIC in opioid self-administration or subsequent opioid seeking, evidence suggests that aIC activity *suppresses* heroin self-administration in some circumstances. Lesions to the aIC made after rats learn to self-administer heroin, but not before, appear to potentiate escalation of heroin self-administration, suggesting that an intact aIC is necessary to maintain control over heroin intake only after acquisition of self-administration [14]. Interestingly, evidence indicates that pre-acquisition aIC lesions increase subsequent escalation of *cocaine* self-administration, whereas post-acquisition aIC lesions decrease subsequent cocaine self-administration [15], unlike the pre- vs post-acquisition effects on heroin self-administration. These findings suggest a more complex role for the IC in regulating drug-related behaviors that depends on prior action-outcome contingency learning and differs across drug type.

Because the IC is interoceptive cortex, it is difficult to disentangle the effects of IC manipulation on drug self-administration from interoceptive processing of the drug itself. Therefore, manipulations during drug seeking without drug reinforcement likely serve as a better measure of IC involvement in drug craving. Indeed, prior work from our laboratory indicates that reversibly inactivating the aIC, but not pIC (referred to there as AId and PIc, respectively), reduces cued reinstatement of cocaine seeking after extinction learning in rats [16]. This finding suggests that, in the absence of drug reinforcement, only the higher-order aIC is critical for expressing the initial action-outcome contingency in response to cocaine-associated external cues. More recent evidence indicates that reversible aIC inactivation also reduces relapse to fentanyl seeking after food choice-induced voluntary abstinence, suggesting a similar role for the aIC in promoting opioid seeking [17, 18]. However, it is unknown whether other opioids, such as heroin, recruit the same aIC mechanisms and whether the pIC is also involved in regulating opioid seeking. To investigate whether these subregions are involved in heroin seeking, the initial experiments in the present study used GABA_B/A_ receptor-based inactivation of the aIC or pIC during reinstatement of heroin seeking after extinction.

However, it is unclear whether the procedures used to suppress drug seeking in rodent models, such as extinction learning, differentially influence aIC or pIC activity during subsequent cued drug seeking. Naqvi and colleagues propose that such competing contingencies selectively recruit the aIC in unreinforced drug seeking [19], although there is a dearth of studies investigating this specific hypothesis. Incubation of craving procedures, wherein rats increase drug seeking in response to drug-associated cues over extended withdrawal periods with no additional contingency learning [20, 21], therefore provide a comparison to extinction-reinstatement procedures. Thus, we followed up the earlier heroin reinstatement work with additional experiments using GABA_B/A_ receptor-based inactivation of the aIC or pIC during a cued incubation of craving test after prolonged withdrawal from heroin.

## Materials and Methods

### Subjects

Male and female Sprague-Dawley rats (250–275 g and 200–250 g, respectively, at time of arrival; Envigo; *n* = 103) were used in this study. The extinction-reinstatement experiments, which were conducted by a former graduate student (CVC) prior to changes in National Institutes of Health policy on sex as a biological variable, used only males, whereas incubation of craving experiments were conducted by a current graduate student (MSM) using both males and females. All rats were single-housed in a temperature-controlled environment under a 12 h light/dark cycle (lights on at 07:00) and allowed to acclimate to the vivarium for at least 2 days before surgery. All procedures followed the National Institutes of Health guidelines for care of laboratory animals and were approved by the University of Iowa Institutional Animal Care and Use Committee.

### Surgery

All rats underwent same-day jugular vein catheter and cranial cannula implant surgeries. Rats were anesthetized with either ketamine (100 mg/kg, i.m.) and xylazine (6 mg/kg, i.m.) or 3–5% isoflurane. Meloxicam (2 mg/kg, s.c.) was administered as an analgesic before surgery as well as 24 h after surgery. Rats also received sterile saline (3 mL, s.c.) after surgery for rehydration.

For catheter implantation, a rounded tip jugular vein catheter (SAI Infusion Technologies) with suture beads 3.0 and 3.5 cm (males) or 2.6 and 3.0 cm (females) from the rounded tip was inserted into the right jugular vein. The opposite end of the catheter was externalized between the shoulder blades and connected to a harness with a 22-gauge guide cannula, which was used for heroin delivery. Catheters were flushed 6 d per week with 0.1 ml of heparinized saline and glycerol to ensure catheter patency. Rats received antibiotics (Baytril, 2.5 m/kg, s.c.) the day of catheter implantation and for 12 days following surgery.

**Fig. 1:**
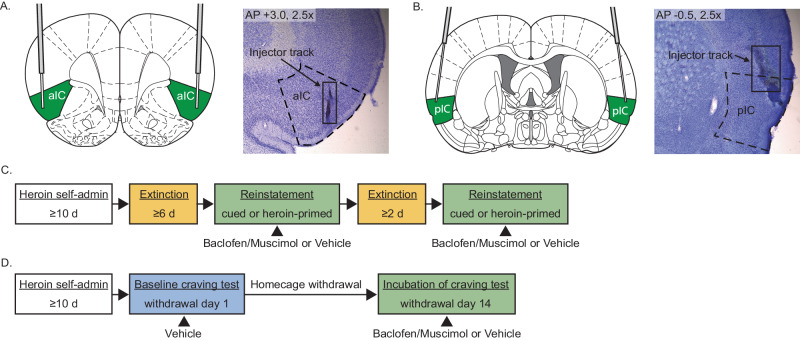
Histology and procedures. **A**, **B** Schematic (*left*) and representative images (*right*) of microinjector termination in the aIC and pIC, respectively. **C** Timeline of extinction-reinstatement procedures. **D** Timeline of incubation of craving procedures.

Rats were then placed in a small animal stereotax (Kopf Instruments), and jeweler’s screws were affixed to the skull surface. Bilateral cannulas (P1 Technologies) were implanted above the aIC (AP + 2.2, ML + 4.5, DV -4.7 at a 2° inward angle) (Fig. 1A) or pIC (AP -1.0, ML + 5.0, DV -4.9 at an 8° outward angle) (Fig. 1B), with all angles with respect to the sagittal plane, and secured with dental cement. Obturators were placed in all cannulas and maintained throughout the experiment. Rats recovered from surgery for at least 5 days before beginning self-administration.

### Heroin self-administration

Rats self-administered heroin 6 days/week in standard operant conditioning chambers, housed within sound-attenuating chambers (Med Associates) and equipped with a central reward magazine flanked by two retractable levers. Cue lights were located directly above both levers, and a 4500 Hz Sonalert pure tone-generator module was positioned above the right lever. A 6 W house light on the opposite wall of the operant chamber was illuminated throughout the training sessions. Heroin (kindly provided by the National Institute on Drug Abuse) was dissolved in 0.9% sterile saline. Concentrations of 0.45 mg/mL heroin for males and 0.34 mg/mL for females were used throughout, with concentrations chosen to produce a dose of approximately 0.075 mg-heroin/kg-body weight per 50 µL infusion for both sexes. A press on the active (right) lever resulted in a 50 µL heroin infusion and a 5 s presentation of light and tone cues. A 20 s timeout period followed each lever press, during which additional active lever presses were recorded but had no scheduled consequence. A press on the inactive (left) lever had no consequence. Rats self-administered heroin in either 3 h (extinction groups, Fig. 1C) or 6 h (withdrawal groups, Fig. 1D) sessions for at least 10 days until criteria were met, with session length chosen based on prior work investigating heroin extinction-reinstatement [22, 23] and incubation of craving [24, 25]. Self-administration completion criteria for 3 h sessions included ≥10 d with ≥10 heroin infusions/day, and ≥10 infusions on each of the final 3 days. Criteria for 6 h sessions included ≥10 days with ≥20 infusions/day, and ≥20 infusions on each of the final 3 days.

### Microinjections

Intra-aIC or intra-pIC microinjections were given immediately before each heroin-seeking test in both extinction-reinstatement and incubation of craving procedures, as well as immediately before open field testing. Microinjectors (with 2 mm and 3 mm projections for the aIC and pIC, respectively) were connected to PE20 tubing, which was attached to 10 µL Hamilton syringes controlled by an infusion pump. The microinjections were 0.2 µL/side, delivered at a rate of 0.3 µL/min. Following each microinjection, microinjectors were left in position for 1 min to allow for diffusion. Immediately following the microinjection, rats were placed into the operant chamber for their appropriate heroin-seeking test. Microinjected drugs consisted of the GABA_B/A_ receptor agonists baclofen and muscimol (BM, given as a cocktail at 1 and 0.1 mM, respectively), dissolved in artificial cerebrospinal fluid (aCSF) as the vehicle, or aCSF vehicle alone. Doses of drugs were chosen based on previous studies [16].

### Extinction-reinstatement procedures

One day after the final self-administration session, rats in the extinction groups proceeded with at least 6 days of 3 h extinction sessions, wherein active lever presses had no consequence. Extinction completion criteria included <30 active lever presses on each of the final 2 days of extinction sessions, with the average of active lever presses on these final 2 days serving as an extinction baseline. After extinction completion criteria were met, rats either underwent cued reinstatement tests or heroin-primed reinstatement tests, but not both. In 3 h cued reinstatement tests, active lever presses produced light and tone cues but no heroin, whereas in 3 h heroin-primed reinstatement tests, active lever presses had no consequence but a priming injection of heroin (0.25 mg/kg s.c.) was given immediately beforehand. Intra-aIC or intra-pIC microinjections of either BM or vehicle were also given immediately beforehand in a within-subjects counterbalanced manner. Each rat underwent its respective reinstatement test twice, once in the BM-treated condition and once in the vehicle-treated condition, with lever pressing re-extinguished to baseline for a minimum of 2 days between counterbalanced reinstatement tests.

### Incubation of craving procedures

One day after the final self-administration session, male and female rats in the withdrawal groups underwent a 30 min cued seeking test, wherein active lever presses produced light and tone cues but no heroin. This test served as the baseline measure for incubation of craving, with the shortened session length chosen to minimize potential extinction learning. Intra-aIC or intra-pIC microinjections of vehicle were given immediately before the baseline test in order to replicate the conditions of the day 14 test. Rats then proceeded with 14 days of homecage withdrawal, followed by a 1 h cued seeking test, because evidence indicates that incubation of heroin craving peaks around day 14 of withdrawal [26, 27]. Intra-aIC or intra-pIC microinjections of either BM or vehicle were given immediately before the day 14 seeking test in a between-subjects manner, with groups selected to have similar levels of day 1 lever pressing on average, and with animals from each group spread across at least three cohorts. The first 30 min of this seeking test was used to assess incubation of craving compared to the day 1 baseline.

### Locomotor activity testing

After completion of incubation of craving procedures, some male and female rats underwent open field testing to determine whether intra-aIC or intra-pIC BM infusions alter locomotor activity in general. Rats received microinjections of either BM or vehicle in a counterbalanced manner (each test separated by 1 d) and were placed into the open field chamber for 30 min. NOLDUS Ethovision recording software was used to record total distance moved during the test.

### Histology

Rats were overdosed with sodium pentobarbital (100 mg/kg, i.p.) and transcardially perfused with 60 mL of PBS (pH 7.4), followed by 60 mL of 4% paraformaldehyde in PBS. Brains were stored in 4% paraformaldehyde for 48 h before sectioning. Brains were coronally sectioned (75 µm) and mounted on gelatin-coated slides to be stained with Cresyl violet. Microinjector termination points were visualized on Cresyl violet-stained sections under a light microscope according to the Paxinos and Watson atlas [28]. Data from any rat whose injection tracks terminated outside the borders of the aIC or pIC were excluded from analysis. Inclusion criteria for the aIC and pIC were identical to those for the AId and PIc, respectively, from our prior work with cocaine seeking [16].

### Statistical analysis

Reinstatement lever pressing data were analyzed using two-way ANOVA with both comparisons as within-subjects repeated measures (extinction baseline vs reinstatement; BM vs vehicle). Incubation lever pressing data from the first 30 min of each seeking test were analyzed using two-way ANOVA with day (day 1 vs day 14) as the within-subjects variable and manipulation (BM vs vehicle) as the between-subjects variable. Where applicable, inactive lever presses, active lever presses, and heroin infusions during the final 10 days of heroin self-administration were also analyzed using two-way ANOVA with day as the within-subjects variable and group (BM vs vehicle) as the between subjects variable To identify potential sex differences in 6 h heroin self-administration, active lever presses, heroin infusions, and bodyweight-adjusted heroin intake during the final 10 sessions were analyzed using two-way ANOVA with day as the within-subjects variable and sex as the between-subjects variable. In all cases, post hoc analyses were completed using Holm–Sidak’s multiple comparisons test. A paired within-subjects *t*-test was used to determine whether the total number of extinction sessions preceding each reinstatement test differed between conditions (BM vs vehicle). Locomotor activity data from the open field test were also analyzed using a paired within-subjects *t*-test. Where applicable, each ANOVA and *t*-test was also run separately for males and females as a preliminary analysis to identify any potential areas where differences may emerge and in accordance with National Institutes of Health policy on sex as a biological variable. *P*-values < 0.05 were considered significant for all analyses. All measures were expressed as mean ± SEM. All data were analyzed using GraphPad Prism 9.4.1 (GraphPad Software).

## Results

### Opposing effects of aIC vs. pIC inactivation on cued reinstatement of heroin seeking after extinction in males

**Fig. 2:**
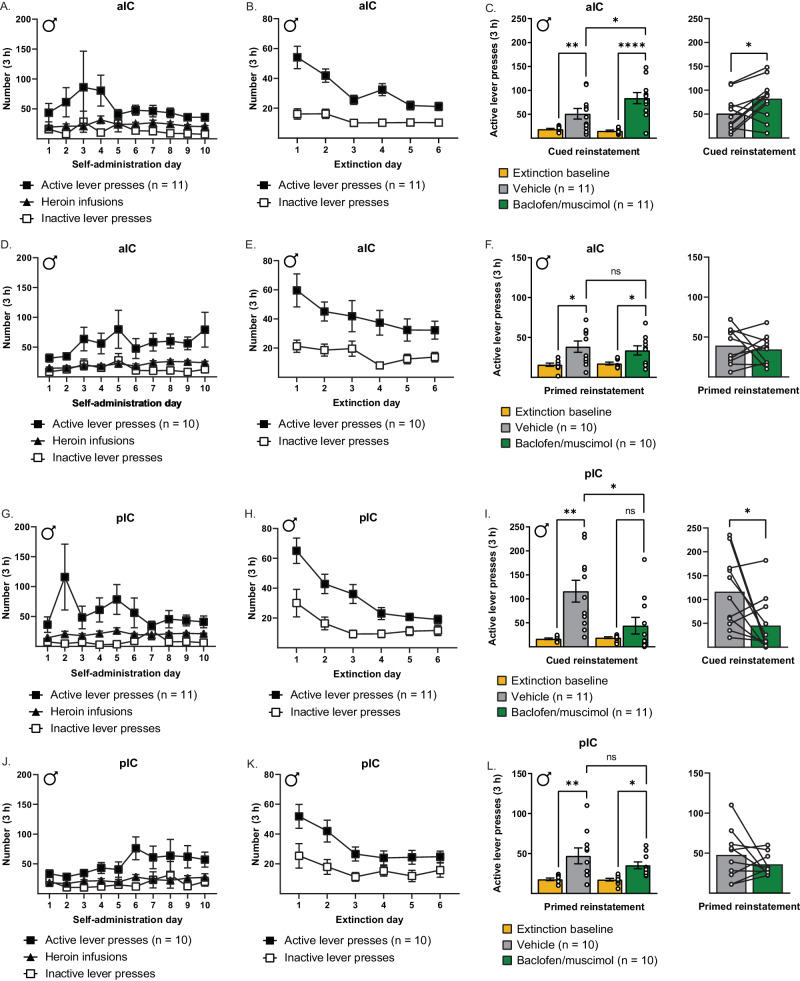
Opposing effects of aIC vs pIC inactivation on cued reinstatement of heroin seeking in males. **A** Lever presses and infusions during the final 10 d of heroin self-administration for rats that would receive aIC injections before cued reinstatement. **B** Lever presses during the first 6 d of extinction for rats that would receive aIC injections before cued reinstatement. **C** Active lever presses during cued reinstatements and extinction baselines (*left*) and within-subjects comparison of reinstatement conditions (*right*). Intra-aIC baclofen/muscimol infusions increased lever pressing during cued reinstatement compared to vehicle controls. **D** Lever presses and infusions during the final 10 d of heroin self-administration for rats that would receive aIC injections before heroin-primed reinstatement. **E** Lever presses during the first 6 d of extinction for rats that would receive aIC injections before heroin-primed reinstatement. **F** Active lever presses during heroin-primed reinstatements and extinction baselines (*left*) and within-subjects comparison of reinstatement conditions (*right*). Intra-aIC baclofen/muscimol infusions had no effect on heroin-primed reinstatement compared to vehicle controls. **G** Lever presses and infusions during the final 10 d of heroin self-administration for rats that would receive pIC injections before cued reinstatement. **H** Lever presses during the first 6 d of extinction for rats that would receive pIC injections before cued reinstatement. **I** Active lever presses during cued reinstatements and extinction baselines (*left*) and within-subjects comparison of individual animals (*right*). Intra-pIC baclofen/muscimol infusions decreased lever pressing during cued reinstatement compared to vehicle controls. **J** Lever presses and infusions during the final 10 d of heroin self-administration for rats that would receive pIC injections before heroin-primed reinstatement. **K** Lever presses during the first 6 days of extinction for rats that would receive pIC injections before heroin-primed reinstatement. **L** Active lever presses during heroin-primed reinstatements and extinction baselines (*left*) and within-subjects comparison of individual animals (*right*). Intra-pIC baclofen/muscimol infusions had no effect on heroin-primed reinstatement compared to vehicle controls. *p < 0.05, **p < 0.01, ***p < 0.001, ****p < 0.0001.

In this experiment, the aIC or pIC was inactivated during either a cued or heroin-primed reinstatement test after extinction to determine the effect on active lever pressing (Fig. 1C). Rats underwent either cued or heroin-primed reinstatement tests (but not both) and received BM or vehicle immediately prior to the tests in a counterbalanced manner. The total number of extinction sessions preceding each reinstatement test did not significantly differ between conditions (Table 1). Figures 2A, B show self-administration and extinction data, respectively, for rats that would receive aIC injections before cued reinstatement. Figure 2C shows active lever presses during cued reinstatement for those rats receiving aIC injections. A two-way repeated measures ANOVA of active lever presses revealed a significant main effect of reinstatement (*F*_1,10_ = 21.35, *p* < 0.001), a significant main effect of manipulation (*F*_1,10_ = 6.60, *p* < 0.05), and a significant interaction (*F*_1,10_ = 7.84, *p* < 0.05). Post hoc tests revealed that rats had increased active lever pressing during cued reinstatement in both the vehicle- and BM-treated conditions compared with extinction baselines (*p* < 0.01; *p* < 0.0001, respectively). However, rats had significantly more active lever presses during cued reinstatement in the BM-treated condition compared to the vehicle-treated condition (*p* < 0.05), indicating that aIC inactivation increased cued reinstatement.

**Table 1:** Total number of extinction sessions before each reinstatement test condition.

Region	Reinstatement	Extinction days before reinstatement (vehicle-treated)	Extinction days before reinstatement (BM-treated)	Paired *t*-test
aIC	Cued	14.18 ± 2.12	12.73 ± 1.42	*t*(10) = 0.48, *p* > 0.15
	Heroin-primed	15.10 ± 1.73	15.40 ± 1.83	*t*(9) = 0.15, *p* > 0.15
pIC	Cued	15.73 ± 2.69	18.91 ± 4.35	*t*(10) = 0.67, *p* > 0.15
	Heroin-primed	13.70 ± 2.34	13.40 ± 1.09	*t*(9) = 0.13, *p* > 0.15

Figures 2D, E show self-administration and extinction data, respectively, for rats that would receive aIC injections before heroin-primed reinstatement. Figure 2F shows active lever presses during heroin-primed reinstatement for those rats receiving aIC injections. A two-way repeated measures ANOVA of active lever presses revealed a significant main effect of reinstatement (*F*_1,9_ = 16.87, *p* < 0.01), but no main effect of manipulation (*F*_1,9_ = 0.12, *p* > 0.15) or interaction (*F*_1,9_ = 0.46, *p* > 0.15). Post hoc tests revealed that, although rats had increased active lever pressing during heroin-primed reinstatement in both the vehicle- and BM-treated conditions compared to extinction baselines (both *p* < 0.05), the vehicle- and BM-treated conditions did not differ in terms of reinstatement of active lever pressing (*p* > 0.15).

Figures 2G, H show self-administration and extinction data, respectively, for rats that would receive pIC injections before cued reinstatement. Figure 2I shows active lever presses during cued reinstatement for those rats receiving pIC injections. A two-way repeated measures ANOVA of active lever presses revealed a significant main effect of reinstatement (*F*_1,10_ = 19.62, *p* < 0.01), a significant main effect of manipulation (*F*_1,10_ = 5.67, *p* < 0.05), and a significant interaction (*F*_1,10_ = 6.51, *p* < 0.05). Post hoc tests revealed that, although rats had increased active lever pressing during cued reinstatement in the vehicle-treated condition compared to extinction baseline (*p* < 0.01), they did not in the BM-treated condition (*p* > 0.15) and this condition had significantly fewer active lever presses compared with the vehicle-treated condition (*p* < 0.05). Thus, pIC inactivation significantly reduced cued reinstatement of active lever pressing.

Figures 2J, K show self-administration and extinction data, respectively, for rats that would receive pIC injections before heroin-primed reinstatement. Figure 2L shows active lever presses during heroin-primed reinstatement for those rats receiving pIC injections. A two-way repeated measures ANOVA of active lever presses revealed a significant main effect of reinstatement (*F*_1,9_ = 15.06, *p* < 0.01), but no main effect of manipulation (*F*_1,9_ = 1.18, *p* > 0.15) or interaction (*F*_1,9_ = 1.37, *p* > 0.15). Post hoc tests revealed that rats had significant reinstatement in both the vehicle- and BM-treated conditions compared to their extinction baselines (*p* < 0.01; *p* < 0.05, respectively), but the vehicle- and BM-treated conditions did not differ in terms of reinstatement of active lever pressing (*p* > 0.15).

### Decreased cued heroin seeking after prolonged withdrawal with pIC, but not aIC, inactivation in males, but not females

**Fig. 3:**
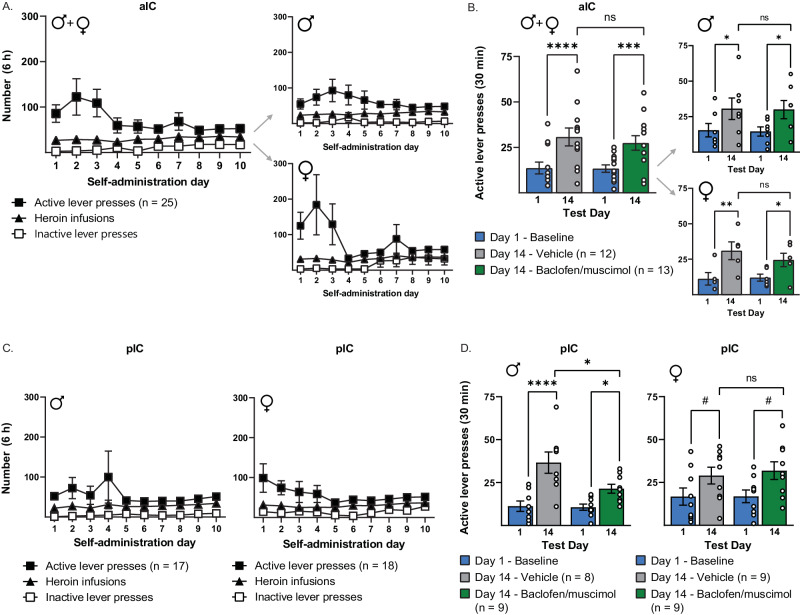
Attenuated incubation of heroin craving with pIC, but not aIC, inactivation in males. **A** Lever presses and infusions during the final 10 d of heroin self-administration for both sexes (*left*), males and females (*right*) in the aIC groups. **B** Intra-aIC baclofen/muscimol infusions had no effect on incubation of heroin craving, as measured by active lever presses during the first 30 min of a day 14 incubation test, compared to vehicle controls in males or females. **C** Lever presses and infusions during the final 10 days of heroin self-administration for males (*left*) and females (*right*) in the pIC groups. **D** Intra-pIC baclofen/muscimol infusions decreased incubation of heroin craving, as measured by active lever presses during the first 30 min of a day 14 incubation test, compared to vehicle controls in males (*left*) but not females (*right*). ^#^*p* < 0.11, *p < 0.05, ****p < 0.0001.

In this experiment, the aIC or pIC was inactivated during a cued heroin-seeking test after 14 days of withdrawal (Fig. 1D). Figure 3A shows self-administration data for the aIC groups, with males and females showing similar levels of lever pressing and heroin infusions during late self-administration days. Two-way ANOVA of inactive lever presses, active lever presses, and heroin infusions during the final 10 d of self-administration revealed no differences between groups that would be treated with BM vs vehicle during the seeking test. (Table 2). Figure 3B shows active lever presses during the day 1 and day 14 cued seeking tests for those rats receiving aIC injections, with injections of vehicle or BM given on day 14 in a between-subjects manner. Analysis of active lever presses during the first 30 min of each seeking test revealed a significant main effect of day (*F*_1,23_ = 51.31, *p* < 0.0001), but no main effect of manipulation (*F*_1,23_ = 0.14, p > 0.15) or interaction (*F*_1,23_ = 0.48, *p* > 0.15). Post hoc tests revealed that both vehicle- and BM-treated groups had increased active lever pressing on day 14 compared to day 1 (*p* < 0.0001; *p* < 0.001, respectively). However, there was no effect of manipulation on day 14 lever pressing (*p* > 0.15), indicating that aIC inactivation did not alter the incubation of heroin craving.

**Table 2:** Statistics for self-administration measures from incubation of craving experiments.

Region	Sex	Effect	Inactive lever	Active lever	Heroin infusions
aIC	M + F	Manipulation	*F*_1,23_ = 0.68, *p* > 0.15	*F*_1,23_ = 0.14, *p* > 0.15	*F*_1,23_ = 1.84, *p* > 0.15
		Day	*F*_1.84,42.34_ = 1.51, *p* > 0.15	*F*_2.32,53.31_ = 1.96, *p* = 0.14	*F*_3.57,82.04_ = 3.26, *p* < 0.05
		Interaction	*F*_9,207_ = 0.51, *p* > 0.15	*F*_9,207_ = 0.30, *p* > 0.15	*F*_9,207_ = 0.61, *p* > 0.15
pIC	M	Manipulation	*F*_1,15_ = 0.42, *p* > 0.15	*F*_1,15_ = 0.31, *p* > 0.15	*F*_1,15_ = 0.23, *p* > 0.15
		Day	*F*_2.41,36.12_ = 2.03, *p* = 0.14	*F*_1.22,18.28_ = 0.80, *p* > 0.15	*F*_1.54,23.03_ = 0.93, *p* > 0.15
		Interaction	*F*_9,135_ = 0.70, *p* > 0.15	*F*_9,135_ = 0.71, *p* > 0.15	*F*_9,135_ = 0.30, *p* > 0.15
	F	Manipulation	*F*_1,16_ = 0.90, *p* > 0.15	*F*_1,16_ = 0.37, *p* > 0.15	*F*_1,16_ = 0.05, *p* > 0.15
		Day	*F*_1.33,21.28_ = 2.53, *p* = 0.12	*F*_2.35,37.60_ = 1.31, *p* > 0.15	*F*_2.43,38.93_ = 3.50, *p* < 0.05
		Interaction	*F*_9,144_ = 1.03, *p* > 0.15	*F*_9,144_ = 0.77, *p* > 0.15	*F*_9,144_ = 0.77, *p* > 0.15

Figure 3C shows self-administration data for the pIC groups. Because sex differences were observed in incubation of craving, both sexes were fully powered, and analyses were performed for each sex separately. Two-way ANOVA of inactive lever presses, active lever presses, and heroin infusions during the final 10 days of self-administration revealed no differences between groups subsequently treated with BM vs vehicle during the seeking test (Table 2). Figure 3D shows active lever presses, separated by sex, during the day 1 and day 14 tests for those rats receiving pIC injections, with injections of vehicle or BM given on day 14 in a between-subjects manner. For the males (left panel), a two-way ANOVA of active lever presses during the first 30 min of each seeking test revealed a significant main effect of day (*F*_1,15_ = 38.38, *p* < 0.0001), a trend toward a main effect of manipulation (*F*_1,15_ = 3.39, *p* = 0.0857), and a significant interaction (*F*_1,15_ = 6.26, *p* < 0.05). Post hoc tests revealed that both vehicle- and BM-treated groups had increased active lever pressing on day 14 compared to day 1 (*p* < 0.0001; *p* < 0.05, respectively). However, the BM group had significantly fewer active lever presses compared to the vehicle group (*p* < 0.05), indicating that pIC inactivation decreased the incubation of heroin craving in males. For the females (right panel), a two-way ANOVA of active lever presses during the first 30 min of each seeking test revealed a significant main effect of day (*F*_1,16_ = 7.24, *p* < 0.05), but no main effect of manipulation (*F*_1,16_ = 0.12, *p* > 0.15) or interaction (*F*_1,16_ = 0.08, *p* > 0.15). Post hoc tests revealed that both vehicle- and BM-treated groups had increased active lever pressing on Day 14 compared to Day 1 that, when separately analyzed, produced non-significant trends (*p* = 0.1070; *p* = 0.1019, respectively). However, there was no effect of manipulation on day 14 lever pressing (*p* > 0.15), indicating that pIC inactivation did not alter the incubation of craving in females.

### No sex differences in 6 h heroin self-administration

**Fig. 4:**
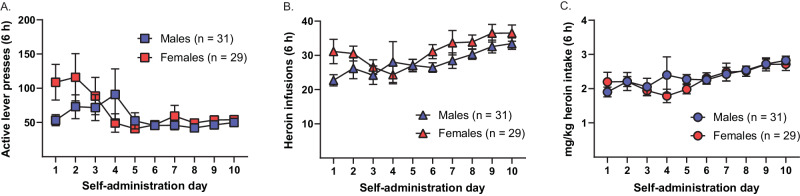
No sex differences in 6-h heroin self-administration. To analyze for potential sex differences in 6 h heroin self-administration, rats were collapsed across the aIC and pIC experiments and the baclofen/muscimol and vehicle groups, and data were analyzed for the final 10 days of self-administration. **A** Daily active lever presses. **B** Daily heroin infusions. **C** Daily heroin intake (mg/kg). There were no significant differences between males and females across the three measures.

We also conducted ancillary analyses of self-administration data, collapsed across the aIC and pIC incubation-of-craving experiments, to determine whether there were any sex differences in 6 h heroin self-administration. Figure 4A–C show, respectively, the total daily active lever presses, total daily heroin infusions, and total daily mg/kg heroin intake across the final 10 days of self-administration for males and females. A two-way ANOVA of active lever presses revealed a significant main effect of day (*F*_3.06,177.30_ = 2.73, *p* < 0.05), but no main effect of sex (*F*_1,58_ = 0.76, *p* > 0.15) and no interaction (*F*_9,522_ = 1.53, *p* = 0.1355). Analysis of heroin infusions revealed a significant main effect of day (*F*_3.38,195.80_ = 4.62, *p* < 0.01), no effect of sex (*F*_1,58_ = 2.13, *p* = 0.1496), and no interaction (*F*_9,522_ = 1.03, *p* > 0.15). When adjusted for body weight, the amount of heroin self-administered did not differ between the sexes, as analysis of mg/kg heroin intake revealed a significant main effect of day (*F*_3.00,174.00_ = 4.49, *p* < 0.01), but no main effect of sex (*F*_1,58_ = 0.19, *p* > 0.15) or interaction (*F*_9,522_ = 0.83, *p* > 0.15).

### No effect of pIC and aIC inactivation on locomotor activity

**Fig. 5:**
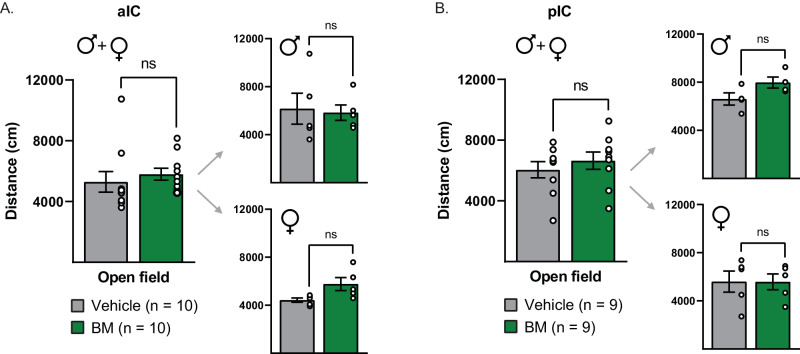
No effect of aIC or pIC inactivation on locomotor activity in either sex. **A** Intra-aIC baclofen/muscimol infusions had no effect on distance traveled in an open field compared to vehicle controls in males or females. **B** Intra-pIC baclofen/muscimol infusions had no effect on distance traveled compared to vehicle controls in males or females.

Figure 5A, B show, respectively, the total distance traveled in an open field test following aIC and pIC inactivation. Paired *t*-tests of total distance travelled revealed no effect of aIC (*t*(9) = 0.94, *p* > 0.15) or pIC (*t*(8) = 0.06, *p* > 0.15) inactivation on locomotor activity in males and females.

## Discussion

The present findings indicate that the aIC and pIC have opposing roles in cued reinstatement of heroin seeking after extinction. aIC and pIC inactivation increased and decreased, respectively, cued reinstatement, yet inactivating either subregion had no effect on heroin-primed reinstatement. In contrast, aIC inactivation after prolonged withdrawal without extinction training had no effect on cued heroin seeking during the day 14 incubation of craving test, whereas pIC inactivation decreased the incubation of craving in males, but not females. These findings suggest that pIC activity is necessary for cued heroin seeking both after extinction training and after prolonged withdrawal in males. However, in females, the pIC does not appear to be involved in cued heroin seeking after prolonged withdrawal. Moreover, the aIC appears to have no role in either sex in cued heroin seeking after prolonged withdrawal.

### Dissociation of aIC and pIC function in reinstatement of drug seeking

Significant work has implicated the IC in promoting drug seeking, although there is evidence of functional dissociation between the aIC and pIC [7]. Previous work from our laboratory indicates that reversible aIC, but not pIC, inactivation reduces cued reinstatement of cocaine seeking after extinction [16], consistent with the idea that competing contingencies selectively recruit the aIC to promote drug seeking [19]. However, no previous work had investigated the IC in cued heroin seeking following self-administration. The present results indicate that aIC inactivation *increased* cued reinstatement of heroin seeking, whereas pIC inactivation reduced cued reinstatement, indicating a dissociation of function both between subregions and for heroin vs cocaine seeking. Although the different effects on cocaine vs heroin seeking are somewhat perplexing, a role for the pIC in promoting opioid-related behaviors is consistent with the limited literature. Indeed, evidence indicates that both inhibiting nitric oxide signaling [9] and blocking muscarinic acetylcholine receptors [10] in the pIC reduce expression of morphine-induced CPP. It appears that the pIC, although generally thought to be important for processing interoceptive cues, is also critical for promoting opioid seeking in response to associated external cues or contexts.

In contrast, the present findings suggest that aIC activity *suppresses* cued reinstatement of heroin seeking following self-administration and extinction training, supporting a more complex role for the aIC in managing drug-related action-outcome contingencies. Prior work indicates that aIC activity also suppresses heroin intake during self-administration [14], although other evidence indicates that post-retrieval aIC manipulations impair subsequent expression of morphine-induced CPP [8]. Nonetheless, passive vs active drug administration differentially alters both brain structure and behavior [29], which may explain the different results. However, our findings also contrast with prior evidence that inactivating the aIC decreases relapse to fentanyl seeking after food choice-induced voluntary abstinence [17, 18]. A role for the aIC in suppressing heroin seeking and self-administration is also in contrast with prior work indicating that aIC activity promotes cocaine seeking [16] and cocaine self-administration [15], further supporting drug type-dependent roles for the aIC in both reinforced drug taking and drug-seeking behavior.

Notably, the present work did not examine the effect of aIC inactivation during a standard extinction test, akin to prior work in other brain regions [30], making it unknown whether the role of the aIC in suppressing heroin seeking is strictly limited to cue-driven heroin seeking. Evidence indicates that both cued and context-induced reinstatement of nicotine seeking are aIC-dependent [31, 32], suggesting that discrete drug-paired cues may not be necessary to recruit the aIC in regulating drug seeking. However, to our knowledge, no prior studies have reported an *increase* in drug or natural reward seeking with aIC inactivation in the absence of cues, and, notably, aIC inactivation did not increase heroin-primed reinstatement in the present study.

### Heterogeneity of IC function across drug types

The differential involvement of the aIC in cocaine vs heroin seeking may reflect the degree to which hedonic vs homeostatic mechanisms control drug seeking [33], differences in strength of interoceptive cues [34, 35], or the competing rewarding and aversive effects of cocaine compared to the more purely rewarding short-term effects of heroin [36, 37]. Moreover, previous work from our laboratory indicates that aIC or pIC inactivation has no effect on reinstatement of food seeking [16], supporting a role for the IC in drug seeking that does not generalize to all forms of reward seeking. The differences between our findings and those with relapse to fentanyl seeking [17, 18] are more perplexing, given that heroin and fentanyl are both opioids; however, these differences could be explained by differential modulation of aIC activity by extinction vs food choice-induced methods to suppress drug seeking.

Nonetheless, studies with other drugs suggest complexity in the role of the aIC, as there is also conflicting evidence on whether aIC activity promotes or suppresses alcohol self-administration [38–40]. Taken together, these findings raise the possibility that there are functionally heterogeneous cell populations within the aIC that promote or suppress drug seeking and that more targeted manipulations would have a greater likelihood of parsing out different effects. Indeed, studies have identified populations of aIC neurons projecting to the nucleus accumbens core and central amygdala that appear to promote alcohol self-administration [40, 41] and methamphetamine seeking [42], and recent evidence indicates that aIC projections to the piriform cortex promote fentanyl seeking [18]. However, these pathways have not yet been investigated in heroin or cocaine seeking. Moreover, no studies have yet identified an aIC pathway that suppresses drug seeking, though human imaging studies indicate a negative correlation between aIC-medial prefrontal cortex resting state functional connectivity and nicotine use and craving [43, 44]. Thus, a distinct possibility is that the activity of aIC projections to a medial prefrontal structure, such as the infralimbic cortex, exerts inhibitory control over heroin seeking in some circumstances.

### Contingency learning and the IC

Critically, the present findings indicate that aIC inactivation had no effect on incubation of heroin seeking, suggesting that new contingency learning, such as extinction, is necessary to recruit this subregion in cued heroin seeking without heroin reinforcement. This is consistent with the hypothesis that drug-associated cues and contexts activate the aIC under conditions in which drug seeking is in conflict with other goals or contingencies [19]. In contrast, pIC inactivation similarly reduced cued heroin seeking after both extinction and prolonged withdrawal in males, indicating that new contingency learning is not necessary to recruit the pIC in heroin seeking without heroin reinforcement. It may be that extinction learning modifies aIC activity to suppress heroin seeking, whereas pIC activity promotes heroin seeking in response to heroin-associated cues regardless of additional contingency learning. As this is the first study to investigate the IC in incubation-of-craving procedures, future work will be needed to determine whether these findings translate to other addictive drugs.

### Sex differences in heroin seeking and IC function

Notably, we found no effect of pIC inactivation on incubation of craving in females. Given the evidence for sex differences in reward systems and risky decision making [45], it is possible that cued heroin seeking after withdrawal in females involves a distinct, pIC-independent mechanism. Moreover, the extinction-reinstatement experiments were conducted only in males, and therefore it is unclear whether there are also sex differences in cued heroin seeking after extinction. The vast majority of extant IC work has been conducted only in male animals, and our results highlight the need for comprehensive comparisons in females. Nonetheless, we found no sex differences in measures of heroin taking in the present study, a finding that is consistent with prior work from our laboratory [24] and others’ [46].

## Conclusion

Together, the present results indicate functional heterogeneity between the aIC and pIC in cued heroin seeking. Notably, aIC inactivation increased cued reinstatement of heroin seeking after extinction, suggesting that aIC activity promotes drug seeking in certain circumstances and pointing to potential heterogeneity of function *within* the aIC. Moreover, inactivating the pIC, but not aIC, reduced cued heroin seeking after prolonged withdrawal in males, indicating that new contingency learning such as extinction may be necessary to recruit the aIC in cued heroin seeking. The present results indicate no effect of pIC inactivation on cued heroin seeking in females, pointing to a potential sex difference. These findings provide some of the first evidence of aIC and pIC regulation of cued heroin seeking and for differential recruitment of the aIC in drug seeking after new contingency learning.

### Supplementary information

Dataset 1

## Data Availability

All data generated or analyzed during this study are included in this public article and its supplementary information files.
